# Novel mutations in *COL4A3*, *COL4A4*, and *COL4A5* in Chinese patients with Alport Syndrome

**DOI:** 10.1371/journal.pone.0177685

**Published:** 2017-05-18

**Authors:** Jian-Hong Liu, Xiu-Xiu Wei, Ang Li, Ying-Xia Cui, Xin-Yi Xia, Wei-Song Qin, Ming-Chao Zhang, Er-Zhi Gao, Jun Sun, Chun-Lin Gao, Feng-Xia Liu, Qiu-Yue Wu, Wei-Wei Li, Asan, Zhi-Hong Liu, Xiao-Jun Li

**Affiliations:** 1 Institute of Clinical Laboratory Science, Jinling Hospital, Nanjing University School of Medicine, Nanjing, China; 2 Binhai Genomics Institute, BGI-Tianjin, BGI-shenzhen, Tianjin, China; 3 National Clinical Research Center of Kidney Diseases, Jinling Hospital, Nanjing University School of Medicine, Nanjing, China; 4 Department of Pediatric Nephrology, Jinling Hospital, Nanjing University School of Medicine, Nanjing, China; 5 State Key Laboratory of Analytical Chemistry for Life Science, Department of Chemistry, Nanjing University, Nanjing, China; Odense University Hospital, DENMARK

## Abstract

Alport syndrome (AS) is a clinically and genetically heterogeneous, progressive nephropathy caused by mutations in *COL4A3*, *COL4A4*, and *COL4A5*, which encode type IV collagen. The large sizes of these genes and the absence of mutation hot spots have complicated mutational analysis by routine polymerase chain reaction (PCR)-based approaches. Here, in order to design a rapid and effective method for the genetic diagnosis of AS, we developed a strategy by utilizing targeted capture associated with next-generation sequencing (NGS) to analyze *COL4A3*, *COL4A4*, and *COL4A5* simultaneously in 20 AS patients. All the coding exons and flanking sequences of *COL4A3*, *COL4A4*, and *COL4A5* from the probands were captured followed by HiSeq 2500 sequencing. Candidate mutations were validated by classic Sanger sequencing and quantitative (q)PCR. Sixteen patients (16/20, 75%) showed X-linked inheritance, and four patients (4/20, 20%) showed autosomal recessive inheritance. None of the individuals had autosomal-dominant AS. Fifteen novel mutations, 6 known mutations, and 2 novel fragment deletions were detected by targeted capture and NGS. Of these novel mutations, 12, 3, and 2 mutations were detected in *COL4A5*, *COL4A4*, and *COL4A3*, respectively. A comparison of the clinical manifestations caused by different types of mutations in *COL4A5* suggested that nonsense mutations and glycine substitution by an acidic amino acid are more severe than the other missense mutations. Pathogenic mutations were detected in 20 patients. These novel mutations can expand the genotypic spectrum of AS. Our results demonstrated that targeted capture and NGS technology are effective in the genetic diagnosis of AS.

## Introduction

Alport Syndrome (AS) is a type of inherited nephropathy characterized by hematuria, proteinuria, and progressive renal failure, often associated with extrarenal manifestations such as sensorineural hypoacusis and ocular abnormalities [1]. The pathogenesis of AS is genetically heterogeneous and is caused by mutations in the genes encoding type IV collagen [2, 3]. The α3, α4 and α5 chains of type IV collagen are encoded by *COL4A3*, *COL4A4*, and *COL4A5*, respectively [4]. Type IV collagen is a major constituent of the glomerular basement membrane (GBM), and is composed of six kinds of homologous α chains, which assemble into three different heterotrimers (α_1_α_1_α_2_, α_3_α_4_α_5_, and α_5_α_5_α_6_) [3]. Among them, the α3α4α5 heterotrimer is essential for the structure and function of the basement membrane in the glomeruli of the kidney, cochlea, and eye [3, 5].

The majority (about 85%) of AS patients show an X-linked dominant inheritance pattern (XLAS, OMIM no. 301050) due to mutations in *COL4A5*, which is located in the Xq22 region [6]; while the other AS patients (about 15%) show autosomal recessive inheritance (ARAS, OMIM no. 104200) and autosomal dominant inheritance (ADAS OMIM no.203780) caused by mutations in *COL4A3* or *COL4A4* located in 2q36.3 [7–11]. *COL4A3*, *COL4A4*, and *COL4A5* are large genes comprising 52, 48, and 53 exons, respectively. Thus far, 225 mutations in *COL4A3*, 170 mutations in *COL4A4*, and 909 mutations in *COL4A5* have been described (The Human Gene Mutation Database at the Institute of Medical Genetics in Cardiff, 2016.4. http://www.hgmd.cf.ac.uk/ac/gene.php?gene=COL4A3; http://www.hgmd.cf.ac.uk/ac/gene.php?gene=COL4A4; http://www.hgmd.cf.ac.uk/ac/gene.php?gene=COL4A5). These mutations are spread throughout the genes without any identified mutational hot spots.

Clinically, AS is heterogeneous, and patients always present a wide variability of clinical manifestations: the rate of progression to end-stage renal disease (ESRD) and the presence or absence of sensorineural deafness and ocular changes [12] depends on the mutation they carry [13–15]. In the case of XLAS, hemizygous males exhibit more severe symptoms than heterozygous female patients and usually reach ESRD before the age of 30 [13]. However, the female patients have more variable symptoms, from isolated hematuria to ESRD [16]. Individuals homozygous for *COL4A3* and *COL4A4* genes resulting in ARAS have similar clinical symptoms and prognosis as males showing XLAS, reaching ESRD in the first or second decade of life [17]. However, early diagnosis and therapy can improve the prognosis. Life expectancy can be increased via early effective and safe therapy for patients with AS [4]. The clinical phenotypes of heterozygous mutations in *COL4A3* and *COL4A4* are heterogeneous, and can cause autosomal dominant AS (ADAS), thin basement membrane nephropathy (TBMN), focal segmental glomerulosclerosis (FSGS) and benign familial hematuria (BFH) [18, 19]. The early clinical manifestations of these diseases are similar, and therefore, genetic classification is critical. However, the large size of the genes and the absence of mutational hot spots have significantly hindered mutational analysis with Sanger sequencing. Recent advances in targeted gene capture associated with next-generation sequencing (NGS) and validated bioinformatics tools have facilitated research and diagnostics in the field of nephrogenetics [20]. Therefore, in this study, we developed a strategy to analyze *COL4A3*, *COL4A4*, and *COL4A5* simultaneously using targeted capture and NGS in 20 patients with AS.

## Materials and methods

### Patients

Twenty patients (18 males and two females) from unrelated Chinese families were elected from 400 patients from 2011 to 2014 diagnosed with AS according to standard criteria, and were enrolled in this study [21]. All these patients met Gregory’s criteria of AS, including family history of nephritis, persistent hematuria and/or proteinuria, immunohistochemical evidence of complete or partial lack of epitope of type IV collagen in glomerular or epidermal basement membranes or both, widespread GBM ultrastructural abnormalities, and presence or absence of ear and eye abnormalities. A brief clinical summary of the patients is shown in Table 1.

**Table 1:** Clinical and pathological features of all the patients.

IID	Sex	Age/AO	URBC (10^4^/ml)	Pro (g/24h)	Scr (mg/dl)	HT	EE	RB			FH
								LM	EM	α3,α5	
1	M	25/24.5	138	6.19	2.32	SH	n.a.	FSGS	L	A,A	P
2	M	15/14	56–225	14.25	0.64	Nor	Nor	FSGS	L	M,M	P
3	M	9/3	1800	1.14	0.60	SH	n.a.	FSGS	L	A,A	P
4	M	25/25	140	5.78	2.64	SH	CC	FSGS	L	M,A	P
5	M	18/18	120	5.46	1.99	SH	n.a.	FSGS	L	M,A	P, C
6	M	21/21	46	4.80	2.43	SH	Nor	FSGS	L	A,A	P
7	M	23/23	46	1.62–1.39	1.07	Nor	Nor	FSGS	L	Nor,M	P
8	M	13/13	110	1.12	0.62	SH	Nor	FSGS	L	M,M	P, C
9	M	15/2	120	6.88	0.89	SH	Nor	FSGS	L	A,A	P
10	M	12/12	15	1.75	0.34	Nor	Nor	FSGS	L	A,A	N
11	F	13/5	640–1200	0.43–1.01	0.54	Nor	Nor	FSGS	L	M,M	P
12	M	23/23	85–226	2.32	0.95	Nor	Nor	FSGS	L	Nor,A	N
13	M	14/11	260	1.79	0.69	Nor	Nor	FSGS	L	A,A	P
14	M	37/26	80	1.13	1.25	SH	n.a	FSGS	L	Nor,M	P
15	M	14/14	490	11.16	2.13	SH	Iso	FSGS	L	A,A	P
16	M	36/32	16	0.71	1.30	Nor	Nor	FSGS	L	A,A	N
17	M	22/16	42	1.22	0.79	n.a.	Nor	FSGS	L	M,M	N
18	M	17/10	77	4.36	1.22	SH	Nor	FSGS	L	A,A	P
19	F	23/2	100	1.22	0.62	n.a.	n.a.	FSGS	L	Nor,Nor	P
20	M	14/10	300	1.71	0.84	SH	CI	FSGS	L	A,A	N

IID, individual ID; AO, age at onset; URBC, urine red blood cell (10^4^/ml); Pro, proteinuria(g/24h); nephritic proteinuria (defined as 24-h urine protein excretion >3.5g); Scr, serum creatinine(mg/dL); normal renal function (defined as SCr<1.24mg/dl); RB, renal biopsy; LM, light microscope; EM, electron microscope; α3,α5, α3,α5 chains in GBM; A, absence; M, mosaic; HT, hearing test; L, lamellation; SH, sensorineural hearing loss; Nor, normal; n.a., not available; EE, eye examination; CC, conjunctival congestion; Iso, Isoametropic; CI, corneal injury; FH, family history; P, positive; N, negative; C: Consanguineous.

Peripheral blood samples were collected from all the participants. This study was approved by the Ethics Committee of Jinling Hospital and written informed consent was obtained from patients and both parents.

### Targeted capture and next-generation sequencing

A custom capture array (NimbleGen, Roche, USA) was designed to capture all exons (1,625), splice sites, and the flanking intron sequences of 78 genes known to be associated with genetic kidney disease, including AS. The total size of the target regions of the capture array was 0.5 Mb. The pipeline described in previous studies [22, 23] was followed to capture and enrich the targeted sequences, prepare the sequencing library, and for NGS. Genomic DNA was extracted from peripheral blood using the QIAamp DNA BloodMiNi Kit (Qiagen, Hilden, Germany) and then sheared into fragments ranging from 200–300 bp using an ultrasonoscope (Covaris S2, Massachusetts, USA). Oligonucleotide adapters from Illumina (single reads) were ligated to the fragments. After the ligation was complete, successful adapter ligation was confirmed by four-cycle PCR using a high-fidelity polymerase with PCR primers containing a custom-synthesized barcode sequence (8 bp) as a sample index signature. PCR was used to generate a library for further analysis, and the DNA adapter-ligated and indexed fragments from 10 libraries were pooled and hybridized to customized oligonucleotide probes. After hybridization of the sequencing primer, base incorporation was carried out on an Illumina Hiseq2500 platform (Illumina, San Diego, CA), following the manufacturer’s standard cluster generation and sequencing protocols, for 90 cycles of sequencing per read, to generate paired-end reads including 90 bps at each end and 8 bps of the index tag by BGI, Tianjin, China.

### Validation of candidate mutations by Sanger sequencing and quantitative (q)PCR

To validate the variations identified with NGS, the corresponding gene regions surrounding the variations were amplified by PCR and sequenced by Sanger sequencing. Purified PCR products were sequenced using the same primers and PCR conditions in both directions on an ABI3730xl DNA sequencer and were analyzed with the sequencer software (Sequencing Analysis 5.2). The two fragment deletions were identified by comparing the normalized sequencing depth of each exon in the same batch. Exons with a depth ratio of specific sample to others > 1.4 were considered to have duplication, while those with values <0.6 were considered to have fragment deletion. The cut-off value for the Z-score was 2.58. qPCR was used to validate the fragment deletions using Step One Plus (Applied Biosystems, CA, USA). The primers used for the amplification of *COL4A3*, *COL4A4*, and *COL4A5* are listed in S1 Table.

### Function prediction and alignment analysis of missense variations

Image analyses, error estimation, and base calling were processed by using the Illumina pipeline (version 1.3.4) after the entire run was completed to generate primary data. Indexed primers were used to identify the different reads from different samples in the primary data, and only reads that were perfectly matched to the theoretical adapter indexed sequences and those that matched the theoretical primer indexed sequences with a maximum of three mismatches were considered acceptable reads. Then, we removed a few unqualified sequences from the primary data using a local dynamic programming algorithm, which included low quality reads, defined as reads that contained >10% Ns in the read length, 50% reads with a quality value of <5 and with an average quality of <10, and adapter sequences including indexed sequences. The remaining sequences were termed as clean reads for further analysis. Clean reads were aligned to the reference human genomic sequence (NCBI37/gh19) of *COL4A3* (NM_000091.4), *COL4A4* (NM_000092.4), and *COL4A5* (NM_033380.2) by using the BWA software package (Burrows Wheeler Aligner) [24]. SNPs and INDELs were identified via the SOAPsnp software and GATK IndelGenotyper (http://www.broadinstitute.org/gsa/wiki/index.php/), respectively [25]. The control database used in the pipeline included the 1000 genome database (http://www.1000genomes.org), dbSNP database, and a BGI in-house database, which included 2,087 normal subjects. Phylop (phyloP46wayPlacental) was used to calculate the conservation of each SNP. The functional impact of missense variation was analyzed with the PolyPhen-2 (Polymorphism Phenotyping v2.2.5) [26] and SIFT algorithm (Sorting Intolerant From Tolerant v5.1) [27]. According to the criteria of the American College of Medical Genetics, we divided the mutation sites into the following five categories: pathogenic, likely pathogenic, benign, likely benign and uncertain significance [28].

## Results

### Identification of candidate mutations in *COL4A3*, *COL4A4*, and *COL4A5*

A total of 272 variants and 2 fragment deletions were identified in *COL4A3*, *COL4A4*, and *COL4A5* from the 20 patients diagnosed with AS using targeted capture and NGS. Among these variants, 90.8% (247/272) were termed as “polymorphisms” with high frequency (>0.01 in control databases). We identified 16 missense mutations, one nonsense mutation, three frameshift mutations, and one 5′-untranslated region (UTR) mutation. Among these, six were known mutations that had been reported previously and 15 were novel ones (Table 2); 66.67% (14/21) were new amino acid substitutions of glycine and 9.52% (2/21) were new amino acid substitutions of proline. These candidate mutations were validated by Sanger sequencing. The frequencies and prediction scores of these mutations are listed in Table 2.

**Table 2:** Mutations detected in *COL4A3*, *COL4A4*, and *COL4A5*.

Sample	Gene Symbol	ExIn_ID	Zygosity	Function	cHGVS	pHGVS	Fr.1^a^	Fr.2^b^	Fr.3^c^	PhyloP Vertebrates	SIFT^d^	HDIV- pph2^e^	HVAR- pph2^f^	Clinical significance [28]	Comment
IID2	COL4A3	EX36	het	missense	c.2990G>A	p.G997E	.	.	.	3.745	D (0)	D (1)	D (1)	pathogenic	Known [29]
IID2	COL4A3	EX40	het	missense	c.3499G>A	p.G1167R	.	.	.	3.689	D (0)	D (1)	D (1)	pathogenic	Known [19]
IID8	COL4A3	EX43	hom	missense	c.3769G>A	p.G1257R	.	.	.	3.71	D (0)	D (1)	D (0.999)	pathogenic	novel
IID10	*COL4A3*	EX44	het	missense	c.3946G>A	p.G1316S	.	.	.	4.445	D (0)	D (1)	D (1)	pathogenic	novel
IID5	COL4A4	EX24	hom	missense	c.1715G>C	p.G572A	.	.	.	3.946	D (0)	D (1)	D (0.998)	pathogenic	novel
IID16	COL4A4	EX24	het	missense	c.1715G>C	p.G572A	.	.	.	3.946	D (0)	D (1)	D (0.998)	pathogenic	novel
IID16	*COL4A4*	.	het	5’UTR	c.-23T>G	.	.	.	.	0.632	NP	NP	NP	US	novel
IID1	*COL4A5*	EX36	hem	missense	c.3179G>A	p.G1060E	.	.	0	4.023	D(0)	D (1)	D (0.985)	pathogenic	novel
IID4	COL4A5	EX26	hem	missense	c.2024G>A	p.G675D	.	.	0	5.288	D (0)	D (1)	D (1)	pathogenic	novel
IID6	COL4A5	EX19	hem	nonsense	c.1117C>T	p.R373*	0	.	0	1.581	-	NP	NP	pathogenic	Known [30]
IID7	COL4A5	EX41	hem	missense	c.3685G>A	p.G1229S	.	.	0	5.481	D (0)	D (1)	D (1)	pathogenic	Known [31]
IID9	COL4A5	EX17	hem	frameshift	c.980_983delATGG	p.D327Vfs*18	.	.	.	.	-	NP	NP	pathogenic	novel
IID11	COL4A5	EX26	het	missense	c.2005G>A	p.G669S	.	.	0	5.374	D (0.01)	D (1)	D (1)	pathogenic	novel
IID12	COL4A5	EX41	hem	missense	c.3685G>A	p.G1229S	.	.	0	5.481	D (0)	D (1)	D (1)	pathogenic	Known [31]
IID13	COL4A5	EX39	hem	missense	c.3509G>T	p.G1170V	.	.	0	4.525	D (0)	D (1)	D (1)	pathogenic	novel
IID14	COL4A5	EX35	hem	missense	c.3088G>A	p.G1030S	0	.	0	5.477	D (0)	D (1)	D (0.999)	pathogenic	Known [32]
IID15	COL4A5	EX31	hem	missense	c.2633G>A	p.G878E	.	.	0	5.635	D (0)	D (1)	D (1)	pathogenic	novel
IID17	COL4A5	EX28	hem	missense	c.2215C>G	p.P739A	0.005425	0.0054	0.0078	1.376	D (0.02)	B(0.338)	B(0.083)	pathogenic	novel
IID18	COL4A5	EX47	hem	frameshift	c.4112delC	p.S1371*	.	.	0	1.917	-	NP	NP	pathogenic	Novel
IID19	COL4A5	EX45	het	missense	c.3958C>T	p.P1320S	.	.	0.0014	2.537	D (0.03)	D (0.956)	D (0.993)	pathogenic	novel
IID20	COL4A5	EX30	hem	frameshift	c.2425_2428delCCAA	p.P809Wfs*9	.	.	.	.	-	NP	NP	pathogenic	novel

^a^Fr.1: Frequency in 1000 genome database

^b^Fr.2: Frequency in dbSNP database

^c^Fr.3: E Frequency in BGI in-house database

^def^Classification: N.P (not predicted), D (Damaging) and B(Benign); US (uncertain significance).

*COL4A3* reference transcript NM_000091.4; *COL4A4* reference transcript NM_000092.4; *COL4A5* reference transcript NM_033380

The criteria of clinical significance are based on American College of Medical Genetics [28].

*: premature stop

In AS patient IID16, in addition to a glycine substitution (G572A) in *COL4A4* in the heterozygous state, there was another heterozygous mutation c.-23T>G in the 5′-UTR of this gene. This heterozygous mutation c.-23T>G was inherited by his two sons (S1 Fig, III1, and III2) who presented persistent isolated hematuria and normal renal function. The clinical features of these two isolated hematuria patients accorded to the diagnosis of familial benign hematuria.

### Identification of fragment deletions in *COL4A5*

**Fig 1:**
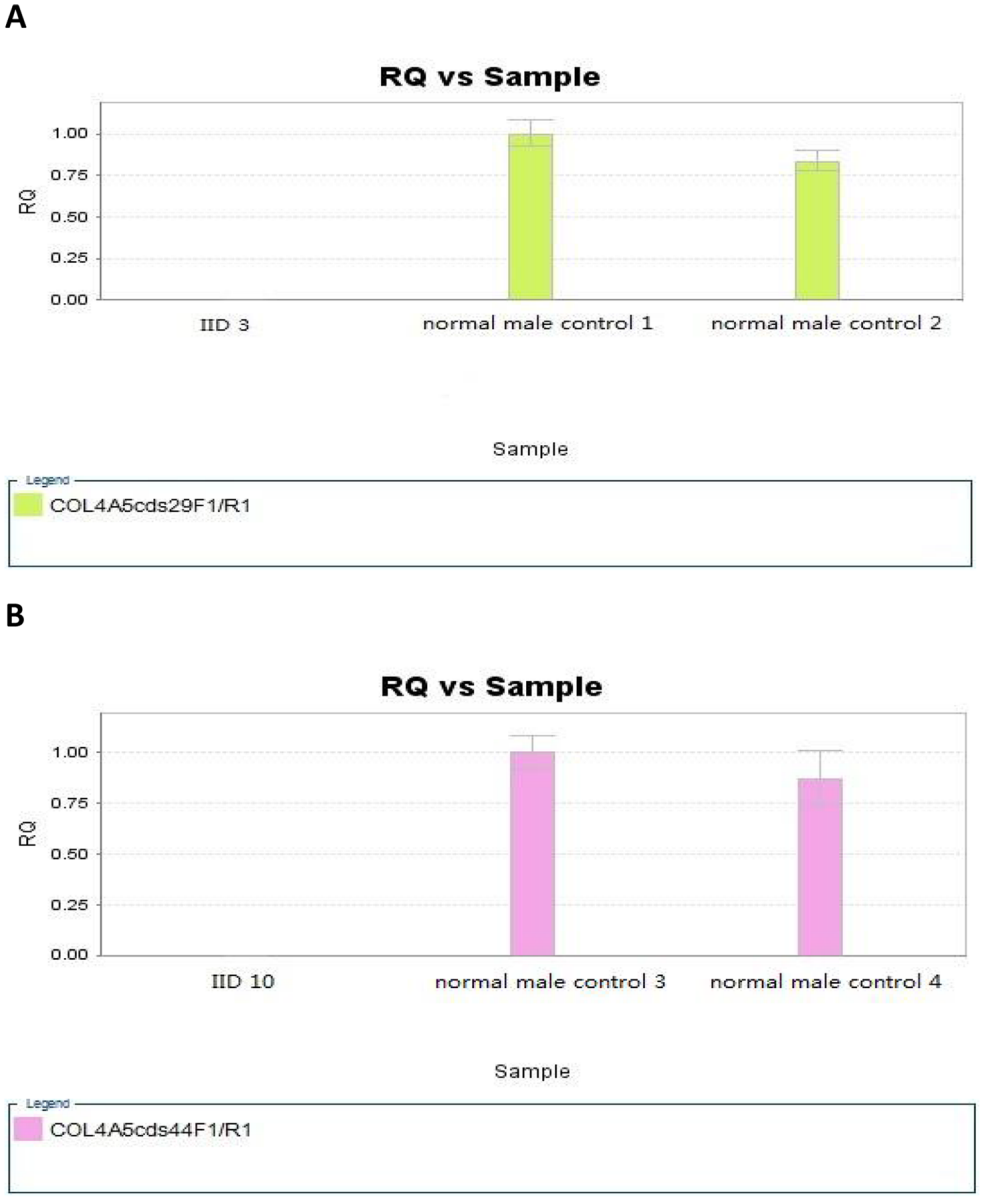
The PCR quantification detected in IID3 and IID10. A, **the PCR quantification results of IID3.** The comparison of quantification of exon 29 of *COL4A5* between patient IID3 and male controls. RQ, real-time quantitative PCR. B, **the PCR quantification results of IID10.** The comparison of quantification of exon 44 of *COL4A5* between patient IID10 and male controls. RQ, real-time quantitative PCR.

IID3 and IID10 carried fragment deletions in *COL4A5* which were validated by qPCR (Fig 1). IID10 had no sensorineural hearing loss and the mildest glomerular injury. However, IID10 carried a heterozygous c.3946G>A (p.G1316S) mutation in *COL4A3* (S2 Fig & Table 2) and a hemizygous deletion of exon 44 *COL4A5*. These two mutations were inherited from each of his parents, who were healthy (S2 Fig). A hemizygous deletion of exon 29 was identified in IID3, and the deletion was inherited from his mother, who had hematuria. The sequencing depths of *COL4A5* exons of patients IID3 and IID10 are listed in S2 Table; the PCR quantification results are shown in Fig 1.

## Discussion

Of the 20 AS patients, 16 (16/20, 75%) showed X-linked inheritance (mutations in *COL4A5*) and four (4/20, 20%) showed autosomal recessive inheritance (mutations in *COL4A3* and *COL4A4*). This ratio is similar to that reported in the literature [4]. In our study, we did not find any individuals with ADAS caused by mutations in *COL4A3* or *COL4A4*. Twenty-one mutations and two fragment deletions were identified by targeted capture and NGS. Sixteen missense mutations, one nonsense mutation, three frameshift mutations, and one 5′-UTR mutation were identified. Among these, 15 were novel mutations and six were known mutations that had been reported previously. According to the criteria of the American College of Medical Genetics [28], the 5′-UTR mutation is an uncertain significant mutation, while the others are pathogenic mutations. Our results showed eight glycine substitutions and two proline substitutions in CO*L4A5*, two glycine substitutions in *COL4A4*, and four glycine substitutions in *COL4A3*. Analyzing the correlations of phenotype and genotype, we found that the clinical phenotypes caused by glycine substitution by an acidic amino acid seem more severe than by a neutral amino acid in *COL4A5*. Patients IID1 (with p.G1060E), IID4 (with p.G675D) and IID15 (with p.G878E) presented relatively more severe symptoms, such as grosser hematuria or proteinuria, higher level of serum creatinine, more rapid progression of disease and with extra renal manifestations (Table 1) than other patients IID7 (with p.G1229S), IID11 (with p.G669S), IID12 (with p.G1229S) and IID13 (with p.G1170V). Similar severity was observed in the clinical phenotypes caused by truncate mutations in *COL4A5*. All male patients IID6 (with p.R373*), IID18 (with p.S1371*) and IID20 (with p.P809Wfs*9) presented extra renal manifestations (Table 1). In addition, IID6 and IID18 had gross hematuria or proteinuria and high-level serum creatinine (Table 1). IID20 exhibited modest clinical manifestations (Table 1), possibly because of his relatively young age. However, as the sample number is not big enough to make a meaningful conclusion, it is necessary to enlarge the sample size for getting further verification in future.

The patient (IID10) with double gene mutations (p.G1316S in *COL4A3* and a hemizygous deletion in *COL4A5*) which were inherited from each of his healthy parents (S2 Fig) had only moderate proteinuria, microscopic hematuria, normal renal function, and no extra renal manifestations. However, Mencarelli *et al* reported that double heterozygotes have more severe phenotypes, such as progression to ESRD by age 44 and sensorineural hearing loss, than individuals with heterozygous mutations in *COL4A5* (in women) or *COL4A4* [33]. As the modest clinical symptoms of IID10, the phenotype of this patient could be caused mainly by the *COL4A5* deletion while *COL4A3* mutation, as an incidental finding, probably played an insignificant role in the patient. However, considering his young age, further follow-up is necessitated for his clinical symptoms.

The individuals with heterozygous mutations of *COL4A3* or *COL4A4* exhibit a broad range of phenotypes. Recent studies have shown that about 40% patients with FBH [34], 10% of familial FSGS patients [35], and <5% AS patients carry heterozygous mutations in *COL4A3* or *COL4A4* [18]. In our study, compound heterozygous mutations p.G997E and p.G1167R in *COL4A3* were identified in the ARAS patient IID2. Interestingly, Xie *et al* reported p.G997E heterozygous mutations in an FSGS patient, and the pathological manifestations showed typical focal segmental glomerulosclerosis [29]. However, the patient IID2, whose renal biopsy showed lamellation changes of the GBM and significantly decreased expression of the α5 chain in GBM, was diagnosed with ARAS. A similar situation was observed in the family of patient IID16. The proband carried two mutations in *COL4A4* (p.G572A and c. -23T>G) and was diagnosed with AS. Both his sons (S1 Fig, III1, and III2) with heterozygous mutations (c. -23T>G) inherited from their father, presented isolated hematuria and accorded to the clinical features of FBH. As the mutations in *COL4A3* or *COL4A4* are heterozygous, half of the α3 or α4 chains in the α3α4α5 heterotrimers of type IV collagen have a normal structure. Therefore, carriers of heterozygous mutations presented mild phenotype clinically. Thus, these carriers are different from the asymptomatic heterozygous carriers of some recessive metabolic diseases such as phenylketonuria. On the basis of these two cases, we proposed that FBH, AS and partial FSGS are type IV collagen-related disorders, and patients who carry a single heterozygous mutation or compound heterozygous mutations may present independent diseases.

Genetic testing is of great and increasing importance for diagnosing AS, and it has some advantages over conventional methods. Compared with renal biopsy, using DNA extracted from peripheral blood for genetic analysis is much less invasive for patients, especially for children. The sequencing results are independent of the stage of disease and the age of the proband. In addition, genetic testing is helpful in clinical diagnosis, especially in sporadic patients or patients whose renal biopsy data not available.

In conclusion, based on targeted capture and NGS, 17 new mutations in genes encoding type IV collagen were identified in 20 AS patients which will be added to the mutation spectrums of *COL4A3*, *COL4A4* and *COL4A5* related to AS. Therefore, combining genetic test with clinical and pathological phenotype, comprehensive diagnosis of AS can be performed at the individual, tissue and molecular levels.

## Supporting information

S1 Fig Pedigree structure of the IID16 family. Squares indicate males and circles indicate females. White symbols indicate individuals without clinical features of the AS disease. Filled gray symbols denote individuals with isolated hematuria or slightly abnormal urine analysis. Filled black symbols denote individuals with hematuria and/or proteinuria, renal failure enven urinaemia. IID, individual ID. The arrows indicate the proband of the family. The horizontal line stand for marriage and double horizontal line stands for consanguineous marriage.
(TIF)

S2 Fig Pedigree structure of the IID3 and IID10 family. (TIF)

S1 Table Primers used in this study. (DOC)

S2 Table The value of sequencing depth of COL4A5 exons of patient IID3 and patient IID10. (DOC)

## References

[1] Alport AC. Hereditary Familial Congenital Haemorrhagic Nephritis. Br Med J. 1927;1(3454):504–6. 20773074 10.1136/bmj.1.3454.504 PMC2454341

[2] Kashtan CE, Michael AF. Alport syndrome: from bedside to genome to bedside. American journal of kidney diseases: the official journal of the National Kidney Foundation. 1993;22(5):627–40. 8238007 10.1016/s0272-6386(12)80424-0

[3] Hudson BG. The molecular basis of Goodpasture and Alport syndromes: beacons for the discovery of the collagen IV family. Journal of the American Society of Nephrology: JASN. 2004;15(10):2514–27. 10.1097/01.ASN.0000141462.00630.76 15466256

[4] Kruegel J, Rubel D, Gross O. Alport syndrome—insights from basic and clinical research. Nature reviews Nephrology. 2013;9(3):170–8. 10.1038/nrneph.2012.259 23165304

[5] Khoshnoodi J, Pedchenko V, Hudson BG. Mammalian collagen IV. Microsc Res Tech. 2008;71(5):357–70. 10.1002/jemt.20564 18219669 PMC4788096

[6] Pirson Y. Making the diagnosis of Alport's syndrome. Kidney international. 1999;56(2):760–75. 10.1046/j.1523-1755.1999.00601.x 10432421

[7] Longo I, Porcedda P, Mari F, Giachino D, Meloni I, Deplano C, et al COL4A3/COL4A4 mutations: from familial hematuria to autosomal-dominant or recessive Alport syndrome. Kidney international. 2002;61(6):1947–56. 10.1046/j.1523-1755.2002.00379.x 12028435

[8] Marcocci E, Uliana V, Bruttini M, Artuso R, Silengo MC, Zerial M, et al Autosomal dominant Alport syndrome: molecular analysis of the COL4A4 gene and clinical outcome. Nephrology, dialysis, transplantation: official publication of the European Dialysis and Transplant Association—European Renal Association. 2009;24(5):1464–71. 10.1093/ndt/gfn681 19129241

[9] Nagel M, Nagorka S, Gross O. Novel COL4A5, COL4A4, and COL4A3 mutations in Alport syndrome. Hum Mutat. 2005;26(1):60. 10.1002/humu.9349 15954103

[10] Savige J, Gregory M, Gross O, Kashtan C, Ding J, Flinter F. Expert guidelines for the management of Alport syndrome and thin basement membrane nephropathy. Journal of the American Society of Nephrology: JASN. 2013;24(3):364–75. 10.1681/ASN.2012020148 23349312

[11] Jefferson JA, Lemmink HH, Hughes AE, Hill CM, Smeets HJ, Doherty CC, et al Autosomal dominant Alport syndrome linked to the type IV collage alpha 3 and alpha 4 genes (COL4A3 and COL4A4). Nephrology, dialysis, transplantation: official publication of the European Dialysis and Transplant Association—European Renal Association. 1997;12(8):1595–9. 10.1093/ndt/12.8.1595 9269635

[12] Wei G, Zhihong L, Huiping C, Caihong Z, Zhaohong C, Leishi L. Spectrum of clinical features and type IV collagen alpha-chain distribution in Chinese patients with Alport syndrome. Nephrology, dialysis, transplantation: official publication of the European Dialysis and Transplant Association—European Renal Association. 2006;21(11):3146–54. 10.1093/ndt/gfl394 16940319

[13] Jais JP, Knebelmann B, Giatras I, De Marchi M, Rizzoni G, Renieri A, et al X-linked Alport syndrome: natural history and genotype-phenotype correlations in girls and women belonging to 195 families: a "European Community Alport Syndrome Concerted Action" study. Journal of the American Society of Nephrology: JASN. 2003;14(10):2603–10. 14514738 10.1097/01.asn.0000090034.71205.74

[14] Gross O, Netzer KO, Lambrecht R, Seibold S, Weber M. Meta-analysis of genotype-phenotype correlation in X-linked Alport syndrome: impact on clinical counselling. Nephrology, dialysis, transplantation: official publication of the European Dialysis and Transplant Association—European Renal Association. 2002;17(7):1218–27. 10.1093/ndt/17.7.1218 12105244

[15] Bekheirnia MR, Reed B, Gregory MC, McFann K, Shamshirsaz AA, Masoumi A, et al Genotype-phenotype correlation in X-linked Alport syndrome. Journal of the American Society of Nephrology: JASN. 2010;21(5):876–83. 10.1681/ASN.2009070784 20378821 PMC2865738

[16] Kashtan CE. Alport syndromes: phenotypic heterogeneity of progressive hereditary nephritis. Pediatric nephrology. 2000;14(6):502–12. 10872195 10.1007/s004670050804

[17] Mochizuki T, Lemmink HH, Mariyama M, Antignac C, Gubler MC, Pirson Y, et al Identification of mutations in the alpha 3(IV) and alpha 4(IV) collagen genes in autosomal recessive Alport syndrome. Nature genetics. 1994;8(1):77–81. 10.1038/ng0994-77 7987396

[18] Kamiyoshi N, Nozu K, Fu XJ, Morisada N, Nozu Y, Ye MJ, et al Genetic, Clinical, and Pathologic Backgrounds of Patients with Autosomal Dominant Alport Syndrome. Clinical journal of the American Society of Nephrology: CJASN. 2016;11(8):1441–9. 10.2215/CJN.01000116 27281700 PMC4974872

[19] Heidet L, Arrondel C, Forestier L, Cohen-Solal L, Mollet G, Gutierrez B, et al Structure of the human type IV collagen gene COL4A3 and mutations in autosomal Alport syndrome. Journal of the American Society of Nephrology: JASN. 2001;12(1):97–106. 11134255 10.1681/ASN.V12197

[20] Renkema KY, Stokman MF, Giles RH, Knoers NV. Next-generation sequencing for research and diagnostics in kidney disease. Nature reviews Nephrology. 2014;10(8):433–44. 10.1038/nrneph.2014.95 24914583

[21] Gregory MC, Terreros DA, Barker DF, Fain PN, Denison JC, Atkin CL. Alport syndrome—clinical phenotypes, incidence, and pathology. Contrib Nephrol. 1996;117:1–28. 8801040 10.1159/000424804

[22] Liu G, Wei X, Chen R, Zhou H, Li X, Sun Y, et al A novel mutation of the SLC25A13 gene in a Chinese patient with citrin deficiency detected by target next-generation sequencing. Gene. 2014;533(2):547–53. 10.1016/j.gene.2013.10.021 24161253

[23] Wei X, Ju X, Yi X, Zhu Q, Qu N, Liu T, et al Identification of sequence variants in genetic disease-causing genes using targeted next-generation sequencing. PLoS One. 2011;6(12):e29500 10.1371/journal.pone.0029500 22216297 PMC3244462

[24] Li H, Durbin R. Fast and accurate short read alignment with Burrows-Wheeler transform. Bioinformatics. 2009;25(14):1754–60. 10.1093/bioinformatics/btp324 19451168 PMC2705234

[25] McKenna A, Hanna M, Banks E, Sivachenko A, Cibulskis K, Kernytsky A, et al The Genome Analysis Toolkit: a MapReduce framework for analyzing next-generation DNA sequencing data. Genome Res. 2010;20(9):1297–303. 10.1101/gr.107524.110 20644199 PMC2928508

[26] Adzhubei IA, Schmidt S, Peshkin L, Ramensky VE, Gerasimova A, Bork P, et al A method and server for predicting damaging missense mutations. Nat Methods. 2010;7(4):248–9. 10.1038/nmeth0410-248 20354512 PMC2855889

[27] Ng PC, Henikoff S. Predicting deleterious amino acid substitutions. Genome Res. 2001;11(5):863–74. 10.1101/gr.176601 11337480 PMC311071

[28] Richards S, Aziz N, Bale S, Bick D, Das S, Gastier-Foster J, et al Standards and guidelines for the interpretation of sequence variants: a joint consensus recommendation of the American College of Medical Genetics and Genomics and the Association for Molecular Pathology. Genet Med. 2015;17(5):405–24. 10.1038/gim.2015.30 25741868 PMC4544753

[29] Xie J, Wu X, Ren H, Wang W, Wang Z, Pan X, et al COL4A3 mutations cause focal segmental glomerulosclerosis. J Mol Cell Biol. 2015;7(2):184 10.1093/jmcb/mjv023 25888712

[30] Pochet JM, Bobrie G, Landais P, Goldfarb B, Grunfeld JP. Renal prognosis in Alport's and related syndromes: influence of the mode of inheritance. Nephrology, dialysis, transplantation: official publication of the European Dialysis and Transplant Association—European Renal Association. 1989;4(12):1016–21. 2517321

[31] Beirowski B, Weber M, Gross O. Chronic renal failure and shortened lifespan in COL4A3+/- mice: an animal model for thin basement membrane nephropathy. Journal of the American Society of Nephrology: JASN. 2006;17(7):1986–94. 10.1681/ASN.2005101044 16775036

[32] Longo I, Scala E, Mari F, Caselli R, Pescucci C, Mencarelli MA, et al Autosomal recessive Alport syndrome: an in-depth clinical and molecular analysis of five families. Nephrology, dialysis, transplantation: official publication of the European Dialysis and Transplant Association—European Renal Association. 2006;21(3):665–71. 10.1093/ndt/gfi312 16338941

[33] Mencarelli MA, Heidet L, Storey H, van Geel M, Knebelmann B, Fallerini C, et al Evidence of digenic inheritance in Alport syndrome. J Med Genet. 2015;52(3):163–74. 10.1136/jmedgenet-2014-102822 25575550

[34] Papazachariou L, Demosthenous P, Pieri M, Papagregoriou G, Savva I, Stavrou C, et al Frequency of COL4A3/COL4A4 mutations amongst families segregating glomerular microscopic hematuria and evidence for activation of the unfolded protein response. Focal and segmental glomerulosclerosis is a frequent development during ageing. PLoS One. 2014;9(12):e115015 10.1371/journal.pone.0115015 25514610 PMC4267773

[35] Malone AF, Phelan PJ, Hall G, Cetincelik U, Homstad A, Alonso AS, et al Rare hereditary COL4A3/COL4A4 variants may be mistaken for familial focal segmental glomerulosclerosis. Kidney international. 2014;86(6):1253–9. 10.1038/ki.2014.305 25229338 PMC4245465

