# Supplementary figures and images for: Novel mutations in COL4A3, COL4A4, and COL4A5 in Chinese patients with Alport Syndrome
Source: PLoS One. 2017 May 18;12(5):e0177685. doi: 10.1371/journal.pone.0177685 (PMC5436713; doi:10.1371/journal.pone.0177685)

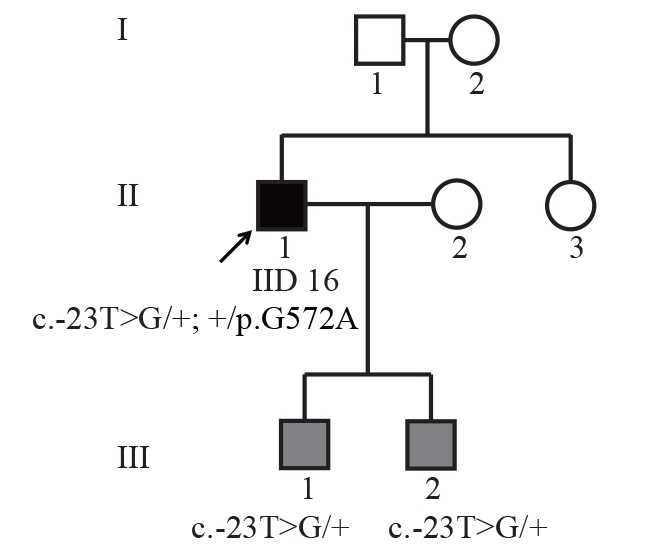

Supplement: S1 Fig — Squares indicate males and circles indicate females. White symbols indicate individuals without clinical features of the AS disease. Filled gray symbols denote individuals with isolated hematuria or slightly abnormal urine analysis. Filled black symbols denote individuals with hematuria and/or proteinuria, renal failure enven urinaemia. IID, individual ID. The arrows indicate the proband of the family. The horizontal line stand for marriage and double horizontal line stands for consanguineous marriage. (TIF) [file pone.0177685.s001.tif]

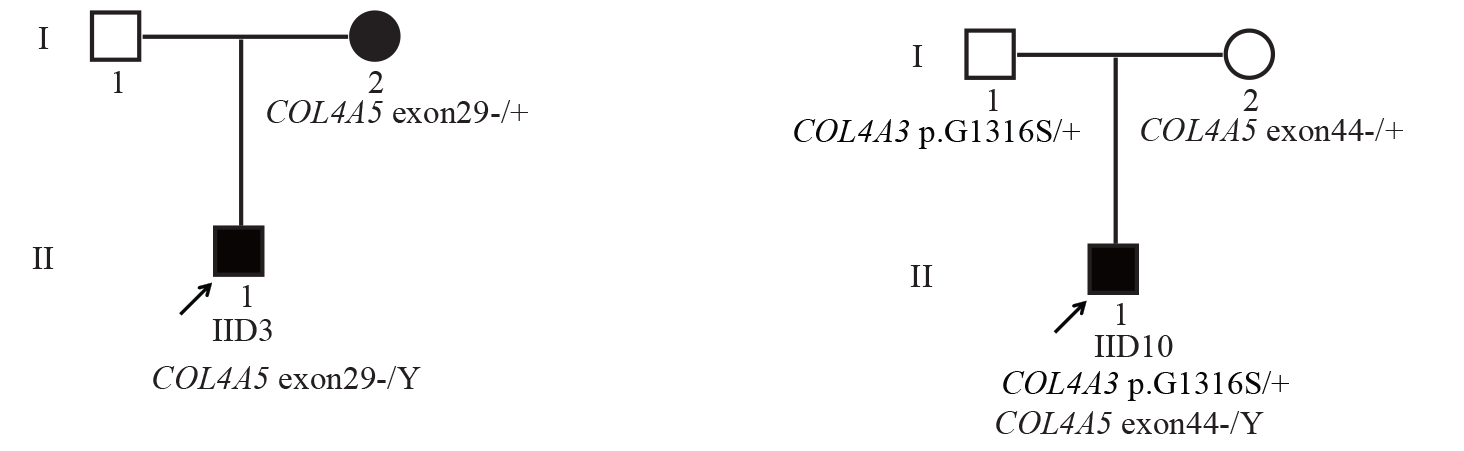

Supplement: S2 Fig — (TIF) [file pone.0177685.s002.tif]
