# Supplementary material for: Novel mutations in COL4A3, COL4A4, and COL4A5 in Chinese patients with Alport Syndrome
Source: PLoS One. 2017 May 18;12(5):e0177685. doi: 10.1371/journal.pone.0177685 (PMC5436713; doi:10.1371/journal.pone.0177685)
Supplement: S1 Table — (DOC) [file pone.0177685.s003.doc]

**Supporting Information**

**Table S1 primers** used in this study

| IID | Mutation Site | Primers |  |
| --- | --- | --- | --- |
| 1 | NM_033380.2(COL4A5):  c.3179G>A (p.G1060E) | F:CAGAAAAGAGGCTGGTGTTGA  R:GACTGGCCACAAACCCTAAA |  |
| 2 | NM_000091.4(COL4A3):  c.2990G>A (p.G997E)  NM_000091.4(COL4A3):  c.3499G>A (p.G1167R) | F:AAACCCAGGTCTGCTAACGA  R:GTGGGCTCATAACAGGACCT  F:ATTCCACACATCTCCCTGGT  R:AGGGGCCCTCAATAAACCTG |  |
| 3 | cds29 deletion in COL4A5 | F:GTGGCAAACAATAAGGACAGAA  R:CAGTGACAGCCTCCATACCT |  |
| 4 | NM_033380.2(COL4A5):  c.2024G>A (p.G675D) | F:AAGGGTAGGAGAGGATGGGT  R:ACCCAGAAGTAGGAGGTTGC |  |
| 5 | NM_000092.4(COL4A4):  c.1715G>C (p.G572A) | F:GGGTGCCAAAAGTGACTCTG  R:GATAGAGTGCTGTGCTTGGC |  |
| 6 | NM_033380.2(COL4A5):  c.1117C>T (p.R373*) | F:GGGACAAGATCAGAGGTGCT  R:TCCTACTTCCATGCTGACCA |  |
| 7 | NM_033380.2(COL4A5):  c.3685G>A (p.G1229S) | F:TCTTCGGGGACACATGACAA  R:ACCTGCCAGCAAAACGAAAT |  |
| 8 | NM_000091.4(COL4A3):  c.3769G>A (p.G1257R) | F:GTGCATGCCTGTAATACCGG  R:GTCTCCCCAGCCATGTAGAA |  |
| 9 | NM_033380.2(COL4A5):  c.980_983delATGG (p.D327Vfs*18) | F:TCATTTGTGCTGATGTCACCC  R:TGCCGATACATGCTCAAAGT |  |
| 10 | NM_000091.4(COL4A3):  c.3946G>A (p.G1315S)  cds44 deletion in COL4A5 | F:TGAATGTGGGAAGCAGAGGT  R:AGGTTACCGAGAGCCATTGA  F:TGTCGTCATTTGCTGTGGAT  R:GGTGGTCCTTGATCTCCTTTC |  |
| 11 | NM_033380.2(COL4A5):  c.2005G>A (p.G669S) | F:AAGGGTAGGAGAGGATGGGT  R:ACCCAGAAGTAGGAGGTTGC |  |
| 12 | NM_033380.2(COL4A5):  c.3685G>A (p.G1229S) | F:TCTTCGGGGACACATGACAA  R:ACCTGCCAGCAAAACGAAAT |  |
| 13 | NM_033380.2(COL4A5):  c.3509G>T (p.G1170V) | F:ACCCTTCGCACCTTACTCTC  R:GGGGAAAGTGTGTGGTAGCT |  |
| 14 | NM_033380.2(COL4A5):  c.3088G>A (p.G1030S) | F:CCATGAAACCAGACAACCCC  R:CCTTTGTCGCCTTTCTGTCC |  |
| 15 | NM_033380.2(COL4A5):  c.2633G>A (p.G878E) | F:TTGTGTGCATGATGTCAAAAGT  R:CGAAGAGGTACCATGGACGA |  |
| 16 | NM_000092.4(COL4A4):  c.1715G>C (p.G572A) | F:GGGTGCCAAAAGTGACTCTG  R:GATAGAGTGCTGTGCTTGGC |  |
| 17 | NM_033380.2(COL4A5):  c.2215C>G (p.P739A) | F:GTGCCTTTCCTTTGGTGGTT  R:TCAGTGGGCAGCCATCAATA |  |
| 18 | NM_000092.4(COL4A4): c.3973+142C>T  NM_033380.2(COL4A5):  c.4112delC (p.S1371*) | F:AGCACTTGAAATGTGGCCAG  R:CCTGGGAGTGTTGACCTTCT  F:GGACAAGCACAGCAAGCAAA  R:GATGGGTTGATAGGTGCAGC |  |
| 19 | NM_033380.2(COL4A5):  c.3958C>T (p.P1320S) | F:CACCTCAGTTAGCCATGGGA  R:ACTTAGCCTCCGATGGTCTG |  |
| 20 | NM_033380.2(COL4A5): c.2425_2428delCCAA (p.P809Wfs*9) | F:TGCTGAATGAATGCCCAGTT  R:TGCTGTACCGCTACTCAGAT |  |
