# Supplementary material for: Novel mutations in COL4A3, COL4A4, and COL4A5 in Chinese patients with Alport Syndrome
Source: PLoS One. 2017 May 18;12(5):e0177685. doi: 10.1371/journal.pone.0177685 (PMC5436713; doi:10.1371/journal.pone.0177685)
Supplement: S2 Table — (DOC) [file pone.0177685.s004.doc]

**Supporting Information**

**Table S2 The value of sequencing depth of COL4A5 exons of patient IID3 and patient IID10.**

| **NO.** | **Gene** | **Exon** | **Depth** | **Sample_normalized**  **_depth** | **others_normalized**  **_depth** | **Sample/others** | **Z-score** |
| --- | --- | --- | --- | --- | --- | --- | --- |
| IID3 | *COL4A5*.NM_033380.2 | EX1 | 82.42 | 0.8436 | 0.983 | 0.8582 | -0.418 |
| IID3 | *COL4A5*.NM_033380.2 | EX2 | 126.1 | 1.2906 | 1.659 | 0.7777 | -0.6891 |
| IID3 | *COL4A5*.NM_033380.2 | EX3 | 118 | 1.2078 | 1.442 | 0.8379 | -0.5387 |
| IID3 | *COL4A5*.NM_033380.2 | EX4 | 125 | 1.2794 | 1.442 | 0.8873 | -0.398 |
| IID3 | *COL4A5*.NM_033380.2 | EX5 | 109.9 | 1.1248 | 1.085 | 1.036 | 0.1806 |
| IID3 | *COL4A5*.NM_033380.2 | EX6 | 115.7 | 1.1842 | 1.297 | 0.913 | -0.2661 |
| IID3 | *COL4A5*.NM_033380.2 | EX7 | 162.4 | 1.6622 | 1.559 | 1.066 | 0.2806 |
| IID3 | *COL4A5*.NM_033380.2 | EX8 | 154.4 | 1.5804 | 1.531 | 1.032 | 0.1083 |
| IID3 | *COL4A5*.NM_033380.2 | EX9 | 156.5 | 1.6018 | 1.58 | 1.014 | 0.0441 |
| IID3 | *COL4A5*.NM_033380.2 | EX10 | 102.4 | 1.0482 | 1.077 | 0.9736 | -0.08299 |
| IID3 | *COL4A5*.NM_033380.2 | EX11 | 100.4 | 1.0276 | 1.054 | 0.975 | -0.08397 |
| IID3 | *COL4A5*.NM_033380.2 | EX12 | 136.6 | 1.3982 | 1.546 | 0.9046 | -0.3243 |
| IID3 | *COL4A5*.NM_033380.2 | EX13 | 123.6 | 1.265 | 1.506 | 0.8398 | -0.4739 |
| IID3 | *COL4A5*.NM_033380.2 | EX14 | 146.9 | 1.5036 | 1.547 | 0.9721 | -0.08547 |
| IID3 | *COL4A5*.NM_033380.2 | EX15 | 122.6 | 1.2548 | 1.477 | 0.8498 | -0.5674 |
| IID3 | *COL4A5*.NM_033380.2 | EX16 | 110.2 | 1.128 | 1.518 | 0.7431 | -0.9194 |
| IID3 | *COL4A5*.NM_033380.2 | EX17 | 129.1 | 1.3214 | 1.503 | 0.8791 | -0.4026 |
| IID3 | *COL4A5*.NM_033380.2 | EX18 | 84.45 | 0.8644 | 1.065 | 0.8113 | -0.5153 |
| IID3 | *COL4A5*.NM_033380.2 | EX19 | 139.4 | 1.4268 | 1.499 | 0.9519 | -0.1626 |
| IID3 | *COL4A5*.NM_033380.2 | EX20 | 116.9 | 1.1966 | 1.32 | 0.9066 | -0.3925 |
| IID3 | *COL4A5*.NM_033380.2 | EX21 | 116.9 | 1.1966 | 1.538 | 0.7781 | -0.7131 |
| IID3 | *COL4A5*.NM_033380.2 | EX22 | 142.8 | 1.4616 | 1.584 | 0.9225 | -0.2829 |
| IID3 | *COL4A5*.NM_033380.2 | EX23 | 97.38 | 0.9968 | 1.352 | 0.7375 | -0.9179 |
| IID3 | *COL4A5*.NM_033380.2 | EX24 | 98.69 | 1.0102 | 1.258 | 0.803 | -0.7002 |
| IID3 | *COL4A5*.NM_033380.2 | EX25 | 122.6 | 1.2548 | 1.284 | 0.9771 | -0.06717 |
| IID3 | *COL4A5*.NM_033380.2 | EX26 | 106 | 1.085 | 1.281 | 0.8469 | -0.6467 |
| IID3 | *COL4A5*.NM_033380.2 | EX27 | 125.4 | 1.2836 | 1.236 | 1.038 | 0.1563 |
| IID3 | *COL4A5*.NM_033380.2 | EX28 | 106.1 | 1.086 | 1.167 | 0.9308 | -0.2349 |
| **IID3** | ***COL4A5*.NM_033380.2** | **EX29** | **0** | **0** | **1.246** | **0** | **-3.342** |
| IID3 | *COL4A5*.NM_033380.2 | EX30 | 122.3 | 1.2518 | 1.406 | 0.8901 | -0.3708 |
| IID3 | *COL4A5*.NM_033380.2 | EX31 | 96.04 | 0.983 | 1.175 | 0.8365 | -0.6511 |
| IID3 | *COL4A5*.NM_033380.2 | EX32 | 123.4 | 1.263 | 1.557 | 0.8112 | -0.4961 |
| IID3 | *COL4A5*.NM_033380.2 | EX33 | 108.3 | 1.1084 | 1.286 | 0.8618 | -0.4388 |
| IID3 | *COL4A5*.NM_033380.2 | EX34 | 122.6 | 1.2548 | 1.399 | 0.8971 | -0.4369 |
| IID3 | *COL4A5*.NM_033380.2 | EX35 | 122.8 | 1.257 | 1.553 | 0.8093 | -0.6502 |
| IID3 | *COL4A5*.NM_033380.2 | EX36 | 107 | 1.0952 | 1.442 | 0.7597 | -0.8404 |
| IID3 | *COL4A5*.NM_033380.2 | EX37 | 116.5 | 1.1924 | 1.376 | 0.8668 | -0.4536 |
| IID3 | *COL4A5*.NM_033380.2 | EX38 | 123.8 | 1.2672 | 1.361 | 0.9314 | -0.2236 |
| IID3 | *COL4A5*.NM_033380.2 | EX39 | 114.3 | 1.17 | 1.364 | 0.8576 | -0.5621 |
| IID3 | *COL4A5*.NM_033380.2 | EX40 | 123.7 | 1.2662 | 1.389 | 0.9116 | -0.3374 |
| IID3 | *COL4A5*.NM_033380.2 | EX41 | 101 | 1.0338 | 1.194 | 0.8657 | -0.4597 |
| IID3 | *COL4A5*.NM_033380.2 | EX42 | 154.7 | 1.5834 | 1.611 | 0.983 | -0.05994 |
| IID3 | *COL4A5*.NM_033380.2 | EX43 | 93.67 | 0.9588 | 1.165 | 0.8232 | -0.6592 |
| IID3 | *COL4A5*.NM_033380.2 | EX44 | 117.5 | 1.2026 | 1.481 | 0.8119 | -0.7122 |
| IID3 | *COL4A5*.NM_033380.2 | EX45 | 122.3 | 1.2518 | 1.335 | 0.9375 | -0.2171 |
| IID3 | *COL4A5*.NM_033380.2 | EX46 | 130.8 | 1.3388 | 1.39 | 0.9628 | -0.156 |
| IID3 | *COL4A5*.NM_033380.2 | EX47 | 114.3 | 1.17 | 1.301 | 0.8992 | -0.3698 |
| IID3 | *COL4A5*.NM_033380.2 | EX48 | 100.7 | 1.0308 | 1.191 | 0.8655 | -0.5091 |
| IID3 | *COL4A5*.NM_033380.2 | EX49 | 134.6 | 1.3776 | 1.38 | 0.9984 | -0.007659 |
| IID3 | *COL4A5*.NM_033380.2 | EX50 | 122.8 | 1.257 | 1.313 | 0.9577 | -0.1541 |
| IID3 | *COL4A5*.NM_033380.2 | EX51 | 138.5 | 1.4176 | 1.63 | 0.8698 | -0.4669 |
| IID3 | *COL4A5*.NM_033380.2 | EX52 | 120.4 | 1.2324 | 1.321 | 0.9329 | -0.27 |
| IID3 | *COL4A5*.NM_033380.2 | EX53E | 148.9 | 1.524 | 1.674 | 0.9105 | -0.3342 |
| IID10 | *COL4A5*.NM_033380.2 | EX1 | 112.4 | 0.9434 | 1.147 | 0.8227 | -0.5205 |
| IID10 | *COL4A5*.NM_033380.2 | EX2 | 163.5 | 1.3722 | 1.585 | 0.8657 | -0.3872 |
| IID10 | *COL4A5*.NM_033380.2 | EX3 | 137.2 | 1.1514 | 1.455 | 0.7912 | -0.6852 |
| IID10 | *COL4A5*.NM_033380.2 | EX4 | 142 | 1.1918 | 1.471 | 0.8103 | -0.5108 |
| IID10 | *COL4A5*.NM_033380.2 | EX5 | 98.51 | 0.8268 | 1.18 | 0.7005 | -0.9107 |
| IID10 | *COL4A5*.NM_033380.2 | EX6 | 136.5 | 1.1456 | 1.37 | 0.8361 | -0.459 |
| IID10 | *COL4A5*.NM_033380.2 | EX7 | 151.6 | 1.2724 | 1.59 | 0.8004 | -0.6181 |
| IID10 | *COL4A5*.NM_033380.2 | EX8 | 149.4 | 1.2538 | 1.599 | 0.7839 | -0.624 |
| IID10 | *COL4A5*.NM_033380.2 | EX9 | 172.7 | 1.4494 | 1.583 | 0.9155 | -0.2478 |
| IID10 | *COL4A5*.NM_033380.2 | EX10 | 114.9 | 0.9644 | 1.284 | 0.751 | -0.7686 |
| IID10 | *COL4A5*.NM_033380.2 | EX11 | 97.25 | 0.8162 | 1.107 | 0.7374 | -0.8668 |
| IID10 | *COL4A5*.NM_033380.2 | EX12 | 158.9 | 1.3336 | 1.547 | 0.862 | -0.3897 |
| IID10 | *COL4A5*.NM_033380.2 | EX13 | 135.4 | 1.1364 | 1.444 | 0.787 | -0.7267 |
| IID10 | *COL4A5*.NM_033380.2 | EX14 | 149 | 1.2506 | 1.706 | 0.7331 | -0.7666 |
| IID10 | *COL4A5*.NM_033380.2 | EX15 | 138.9 | 1.1658 | 1.512 | 0.771 | -0.6691 |
| IID10 | *COL4A5*.NM_033380.2 | EX16 | 145.1 | 1.2178 | 1.47 | 0.8284 | -0.6019 |
| IID10 | *COL4A5*.NM_033380.2 | EX17 | 162.5 | 1.3638 | 1.513 | 0.9016 | -0.318 |
| IID10 | *COL4A5*.NM_033380.2 | EX18 | 132.6 | 1.1128 | 1.113 | 0.9995 | -0.001362 |
| IID10 | *COL4A5*.NM_033380.2 | EX19 | 163.4 | 1.3714 | 1.444 | 0.9497 | -0.1485 |
| IID10 | *COL4A5*.NM_033380.2 | EX20 | 146.7 | 1.2312 | 1.523 | 0.8082 | -0.5818 |
| IID10 | *COL4A5*.NM_033380.2 | EX21 | 132.2 | 1.1096 | 1.445 | 0.7677 | -0.6467 |
| IID10 | *COL4A5*.NM_033380.2 | EX22 | 178.2 | 1.4956 | 1.591 | 0.94 | -0.1575 |
| IID10 | *COL4A5*.NM_033380.2 | EX23 | 126.4 | 1.0608 | 1.205 | 0.8805 | -0.3563 |
| IID10 | *COL4A5*.NM_033380.2 | EX24 | 129.7 | 1.0886 | 1.467 | 0.742 | -0.7441 |
| IID10 | *COL4A5*.NM_033380.2 | EX25 | 151 | 1.2674 | 1.484 | 0.8542 | -0.426 |
| IID10 | *COL4A5*.NM_033380.2 | EX26 | 128.2 | 1.076 | 1.369 | 0.7858 | -0.6865 |
| IID10 | *COL4A5*.NM_033380.2 | EX27 | 154.6 | 1.2976 | 1.392 | 0.9323 | -0.2323 |
| IID10 | *COL4A5*.NM_033380.2 | EX28 | 114.1 | 0.9576 | 1.248 | 0.7675 | -0.6558 |
| IID10 | *COL4A5*.NM_033380.2 | EX29 | 124.6 | 1.0458 | 1.374 | 0.7614 | -0.7141 |
| IID10 | *COL4A5*.NM_033380.2 | EX30 | 139.9 | 1.1742 | 1.4 | 0.8387 | -0.4932 |
| IID10 | *COL4A5*.NM_033380.2 | EX31 | 135.1 | 1.1338 | 1.407 | 0.8059 | -0.5893 |
| IID10 | *COL4A5*.NM_033380.2 | EX32 | 156.2 | 1.311 | 1.652 | 0.7935 | -0.6664 |
| IID10 | *COL4A5*.NM_033380.2 | EX33 | 140.9 | 1.1826 | 1.444 | 0.819 | -0.4645 |
| IID10 | *COL4A5*.NM_033380.2 | EX34 | 158 | 1.326 | 1.473 | 0.9003 | -0.2773 |
| IID10 | *COL4A5*.NM_033380.2 | EX35 | 167.7 | 1.4074 | 1.657 | 0.8491 | -0.4502 |
| IID10 | *COL4A5*.NM_033380.2 | EX36 | 153.3 | 1.2866 | 1.515 | 0.8493 | -0.4319 |
| IID10 | *COL4A5*.NM_033380.2 | EX37 | 156.3 | 1.3118 | 1.39 | 0.9439 | -0.1605 |
| IID10 | *COL4A5*.NM_033380.2 | EX38 | 145.1 | 1.2178 | 1.484 | 0.8208 | -0.5256 |
| IID10 | *COL4A5*.NM_033380.2 | EX39 | 146.5 | 1.2296 | 1.504 | 0.8174 | -0.6553 |
| IID10 | *COL4A5*.NM_033380.2 | EX40 | 126.9 | 1.065 | 1.356 | 0.7857 | -0.6521 |
| IID10 | *COL4A5*.NM_033380.2 | EX41 | 127 | 1.0658 | 1.454 | 0.7328 | -0.8865 |
| IID10 | *COL4A5*.NM_033380.2 | EX42 | 137.4 | 1.1532 | 1.549 | 0.7442 | -0.8043 |
| IID10 | *COL4A5*.NM_033380.2 | EX43 | 113.8 | 0.955 | 1.066 | 0.8958 | -0.3051 |
| **IID10** | ***COL4A5*.NM_033380.2** | **EX44** | **0** | **0** | **1.53** | **0** | **-2.761** |
| IID10 | *COL4A5*.NM_033380.2 | EX45 | 139.2 | 1.1682 | 1.499 | 0.7795 | -0.6442 |
| IID10 | *COL4A5*.NM_033380.2 | EX46 | 131 | 1.0994 | 1.449 | 0.7589 | -0.7177 |
| IID10 | *COL4A5*.NM_033380.2 | EX47 | 153.7 | 1.29 | 1.361 | 0.9479 | -0.1493 |
| IID10 | *COL4A5*.NM_033380.2 | EX48 | 128.4 | 1.0776 | 1.209 | 0.8914 | -0.304 |
| IID10 | *COL4A5*.NM_033380.2 | EX49 | 141 | 1.1834 | 1.431 | 0.8271 | -0.4926 |
| IID10 | *COL4A5*.NM_033380.2 | EX50 | 127.8 | 1.0726 | 1.405 | 0.7636 | -0.7232 |
| IID10 | *COL4A5*.NM_033380.2 | EX51 | 133.5 | 1.1204 | 1.536 | 0.7295 | -0.8503 |
| IID10 | *COL4A5*.NM_033380.2 | EX52 | 159.2 | 1.3362 | 1.398 | 0.9557 | -0.1326 |
| IID10 | *COL4A5*.NM_033380.2 | EX53 | 145.9 | 1.2246 | 1.564 | 0.7828 | -0.6608 |
